# Shenzhiling Oral Liquid Protects STZ-Injured Oligodendrocyte through PI3K/Akt-mTOR Pathway

**DOI:** 10.1155/2020/4527283

**Published:** 2020-07-24

**Authors:** Zhenhong Liu, Gaofeng Qin, Lulu Mana, Shuaiyang Huang, Yahan Wang, Pengwen Wang

**Affiliations:** ^1^Key Laboratory of Chinese Internal Medicine of Ministry of Education and Beijing, Dongzhimen Hospital, Beijing University of Chinese Medicine (BUCM), Beijing, China; ^2^Key Laboratory of Pharmacology of Dongzhimen Hospital (BUCM), State Administration of Traditional Chinese Medicine, Beijing, China; ^3^Xinjiang Medical University, Urumqi, China

## Abstract

White matter degeneration and demyelination are nonnegligible pathological manifestations of Alzheimer's disease (AD). The damage of myelin sheath consisting of oligodendrocytes is the basis of AD's unique early lesions. Shenzhiling oral liquid (SZL) was the effective Chinese herbal compound approved by the Food and Drug Administration (FDA) for the treatment of AD in China, which plays the exact therapeutic role in clinical AD patients. However, its molecular mechanism remains unclear to date. For this purpose, an in vitro mode of streptozotocin- (STZ-) induced rat oligodendrocyte OLN-93 cell injury was established to mimic the pathological changes of myelin sheath of AD and investigate the mechanism of SZL protecting injured OLN-93 cell. The results showed that STZ can decrease cell viability and downregulate the activity of PI3K/Akt-mTOR signalling pathway and the expression of myelin sheath-related proteins (MBP, MOG, and PLP) in OLN-93 cells. Both SZL-medicated serum and donepezil (positive control) can protect cells from STZ-caused damage. SZL-medicated serum increased OLN-93 cell viability in a dose- and time-dependent manner and enhanced the activity of PI3K/Akt-mTOR signalling pathway. The inhibitor of PI3K (LY294002) inhibited the protective effect of SZL-medicated serum on the STZ-injured OLN-93 cells. Furthermore, rapamycin, the inhibitor of mTOR, inhibited the promotion of cell viability and upregulation of p-mTOR and MBP caused by SZL-medicated serum. In conclusion, our data indicate that SZL plays its therapeutic role on AD by promoting PI3K/Akt-mTOR signalling pathway of oligodendrocytes. Thus, the present study may facilitate the therapeutic research of AD.

## 1. Introduction

Alois Alzheimer proposed in 1911 that the onset of Alzheimer's disease (AD) was accompanied by myelin destruction and intracellular lipid deposits [1]. The loss of myelin integrity in AD patients even preceded the onset of cognitive impairment [2]. White matter degeneration and demyelination were not negligible pathological manifestations of AD in addition to classical nerve cell injury.

Oligodendrocytes (OLs) differentiated from oligodendrocyte precursor cells (OPCs) can wrap around neuronal axons to form myelin sheath, assist the myelin sheath in completing transmission of electrical signals between nerves, provide nutritional support for neuronal axons [3], and maintain normal physiological functions of neurons. Oligodendrocytes played a significant role in the pathological process of AD [4]. The processes of myelin formation and myelin regeneration were strictly regulated by multiple coordinated signal transduction pathways, such as the Wnt/*β*-catenin, PI3K/Akt-mTOR, and ERK/MAPK pathways. Among them, the PI3K/Akt-mTOR pathway plays an important role in regulating the differentiation of oligodendrocytes and the formation of myelin sheath [5].

Shenzhiling oral liquid (SZL) was the Chinese medicine compound approved by the Chinese Food and Drug Administration (CFDA) for the treatment of AD in China. SZL contains ten traditional Chinese medicines: *Codonopsis pilosula*, *Cinnamomum cassia Presl*, *Cynanchum otophyllum* Schneid, *Glycyrrhizae*, *Poria cocos*, *Gried ginger*, *Polygala tenuifolia* Willd., *Acorus tatarinowii*, *Dragon bone,* and *Ostrea gigas* Thunberg. SZL has positive clinical effect on AD, such as improving scores on the minimental state examination (MMSE), the Montreal cognitive assessment scale (MoCA), the Alzheimer's disease assessment scale-cognitive portion (adas-cog), and the operational capacity in both hands [6]. In addition, SZL delayed the progression of cognitive and behaviour symptoms in AD patients to some extent [7].

Previous studies by our research group confirmed that SZL can improve synaptic plasticity and, thus, improve the learning and memory ability of APP^swe^/PSl^dE9^ mice [8]. However, more in-depth studies should be conducted on the mechanism of SZL on the structure and function of oligodendrocytes and myelin sheath in early AD. PI3K/Akt-mTOR signalling pathway plays critical role in oligodendrocyte proliferation, survival, and differentiation. Thus, we speculated that SZL might play its therapeutic role in AD by regulating PI3K/Akt-mTOR pathway. Here we proved this speculation by investigating the role of SZL in regulating myelination of AD cell model, which may facilitate the clinical application of SZL in the treatment of AD.

## 2. Materials and Methods

### 2.1. Drug Preparation

SZL was purchased from Shandong Wohua Pharmaceutical Co., Ltd. (SFDA Approval No. Z20120010), which was dissolved in water to a final concentration of 40% (v/v). DMSO (D2650, Sigma-Aldrich, St. Louis, MO, USA) was used to prepare 10 mM stock solution of donepezil (D6821, Sigma-Aldrich, St. Louis, MO, USA), which was filtered and preserved at −20°C. DMEM (SH30022.01, Hyclone, Logan, Utah, USA) was used to prepare 10 mM stock solution of streptozotocin (STZ) (S0130, Sigma-Aldrich, St. Louis, MO, USA). Before the experiment, the stock solution was diluted with DMEM to the required concentrations.

### 2.2. Preparation of SZL-Medicated Serum

Male SD rats (200 ± 20 g) were purchased from Beijing Vital River Laboratory Animal Technology Co., Ltd. (Certificate SCYK2016-0006, Beijing, China) and were kept in the barrier environment animal room of Dongzhimen Hospital of Beijing University of Chinese Medicine (Certificate SCYK2015-0001, Beijing, China). They were raised in a single cage with free food and drinking water, and the bedding material was changed regularly. Forty Sprague Dawley (SD) rats were randomly divided into the normal group (*n* = 10) and the SZL group (*n* = 30). In the SZL group, 40% Shenzhiling oral liquid (an amount twice the clinical equivalent dose for a 70 kg adult) was administered intragastrically once a day at 8 : 00 am for 7 days, and the normal group was administered an equal volume of water. The dosage and the duration of SZL treatment are used here according to our previous experiments (data not shown) and relevant literatures [9–13]. On the 7th day, 1 h after the last administration, the rats were anesthetized by intraperitoneal injection of 1.5% sodium pentobarbital and sacrificed with excessive sodium pentobarbital. The blood was collected from the abdominal aorta using a disposable blood sampling needle and vacuum blood collection. After the blood was taken into the vacuum blood vessels, the blood vessels were placed at 4°C for 1 h, centrifuged at 3500 rpm for 10 min, and separated. The separated serum was mixed well, inactivated at 56°C for 30 min in a thermostat water bath, packaged, and frozen at −80°C for later use. All experiments were performed in compliance with Beijing's regulations and guidelines for the use of animals in research, and the study was approved by the Animal Research Ethics Board of Dongzhimen Hospital.

### 2.3. Determination of the Main Chemical Constituents in SZL-Medicated Serum by UHPLC-MRM-MS/MS Analysis

Serum samples were thawed at 4°C and centrifuged at 12000 rpm for 10 min at 4°C. Then, 160 *μ*l of methanol was added to a centrifugal tube containing 40 *μ*l of sample. The sample was vortexed for 30 seconds and sonicated for 5 min three times. After standing at −20°C for 1 h, the sample was centrifuged at 1°C for 1 min at 12,000 rpm. Finally, the clear supernatant was placed in the autosampler vial for LC-MS/MS analysis. The target compound was chromatographed on a Waters ACQUITY UPLC HSS T3 column (100 × 2.1 mm, 1.8 *μ*m) using an Agilent 1290 Infinity II series UHPLC System (Agilent Technologies). Mobile phase A was 0.1% acetic acid in water, and mobile phase B was methanol. The column temperature was set at 35°C. The autosampler temperature was set at 4°C, and the injection volume was 1 *μ*l. Mass spectrometry was performed in multiple reaction monitoring (MRM) mode using an Agilent 6460 triple quadrupole mass spectrometer (Agilent Technologies) equipped with an AJS electrospray ionization (AJS-ESI) interface. The ion source parameters were a capillary voltage of +4000/−3500 V, a nozzle voltage of +500/−500 V, a gas (N_2_) temperature of 300°C, a gas (N_2)_ flow of 5 L/min, a sheath gas (N_2_) temperature of 250°C, a sheath gas flow of 11 L/min, and a nebulizer of 45 psi. MRM data acquisition and processing were performed by Agilent Mass Hunter Work Station Software (B.08.00, Agilent Technologies).

### 2.4. Cell Culture

Rat oligodendrocyte OLN-93 cells were a gift from Professor Zhicheng Xiao of Monash University, Australia. Experiments were performed using 30 to 40 generations of cells. OLN-93 cells were cultured in high glucose Dulbecco's modified Eagle's medium (DMEM) containing 10% foetal bovine serum (FBS) and 1% penicillin/streptomycin in a 37°C, 5% CO_2_ atmosphere.

### 2.5. Hematoxylin and Eosin (H&E) Staining

OLN-93 cells in each group were stained to observe cell morphology according to the instruction of the haematoxylin and eosin (H&E) staining kit (G1120, Solarbio, Beijing, China).

### 2.6. Cell Viability/Cytotoxicity Assay

Cell viability and toxicity were measured following the instruction of the Viability/Cytotoxicity Multiplex Assay Kit (CK17, DOJINDO, Shanghai, China).

### 2.7. Western Blot

Total cell protein was extracted using highly efficient RIPA tissue/cell lysate (R0010, Solarbio, Beijing, China) containing 1% PMSF and 1% protein phosphatase inhibitor (P1260, Solarbio, Beijing, China) and carefully following the corresponding instruction. Cell protein concentration was measured with the bicinchoninic acid (BCA) method. First, 20 *µ*g of cell protein was loaded into each well, and proteins of different molecular weights were separated by SDS-polyacrylamide gel electrophoresis (SDS-PAGE). The desired gels were cut according to the molecular weight of target proteins and transferred onto the polyvinylidene fluoride (PVDF) membranes. Then, the PVDF membranes were blocked with 5% (w/v) nonfat dry milk for 1 h before incubating in a primary antibody for PI3K (1 : 1000, ab74136, Abcam, USA), Akt (1 : 1000, ab8805, Abcam, USA), p-Akt (1 : 1000, 4060, Cell Signalling Technology, USA), mTOR (1 : 1000, 2983, Cell Signalling Technology, USA), p-mTOR (1 : 1000, 5536, Cell Signalling Technology, USA), MBP (1 : 1000, 78896, Cell Signalling Technology, USA), MOG (1 : 1000, ab32760, Abcam, USA), PLP (1 : 2000, ab28486, Abcam, USA), and *β*-actin (1 : 8000, Abcam, USA) at 4°C overnight. PVDF membranes continued to incubate for 1 h with appropriate HRP-conjugated secondary antibodies (1 : 8000, ZSGQ-BIO, Beijing, China). Finally, bands were detected with ECL, and the results were analysed using Image J gel analysis software.

### 2.8. Gene Expression Assessment

Total RNA extraction was performed according to the instruction of the RNAprep Pure Cell/Bacteria Kit (DP430, TIANGEN BIOTECH (Beijing) CO., LTD, China). Reverse transcription was performed according to the instruction of the FastKing gDNA Dispelling RT SuperMix (KR118, TIANGEN BIOTECH (Beijing) CO., LTD, China). The primer sequences are shown in Table 1. QPCR was performed according to the instruction of the Talent qPCR PreMix (SYBR Green) (FP209, TIANGEN BIOTECH (Beijing) CO., LTD., China). The relative transcript level of the target gene was calculated using the 2^−ΔΔCT^ method.

### 2.9. Statistical Analysis

SPSS22.0 statistical analysis software was used for statistical analysis of all experimental data. If the variances were equal and *n* ≥ 6, one-way ANOVA was used to determine differences among the groups that were statistically significant. Data are described as the mean ± standard deviation (SD). Otherwise, the nonparametric test was used. Mann–Whitney *U* test was used to determine differences between two independent groups. The Tukey's multiple comparison was used for post hoc test after one-way ANOVA and Dunn's multiple comparisons test was used after Kruskal–Wallis H test. Data are described as the median (IQR). IQR means interquartile range. *P* < 0.05 was considered statistically significant.

## 3. Results

### 3.1. SZL-Medicated Serum Contained Paeoniflorin, Liquiritin, Cinnamic Acid, and Glycyrrhizic Acid by UHPLC-MRM-MS/MS Analysis

**Figure 1:**
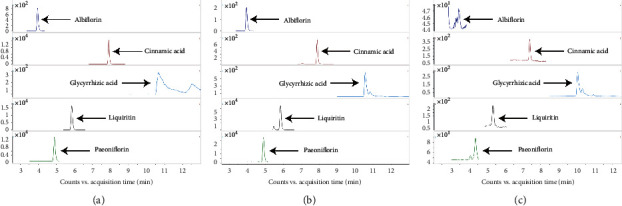
Extracted ion chromatographs from standard solution (a), SZL (b), and SZL-medicated serum (c).

Cinnamic acid and paeoniflorin were the quality control indexes of the quality inspection standards for SZL [14, 15]. To detect the metabolic components in the SZL-medicated serum, we chose UHPLC-MRM-MS/MS for quantitatively analysing the metabolites of albiflorin, paeoniflorin, liquiritin, cinnamic acid, and glycyrrhizic acid. The selection of these five compounds was based on relevant literature [16, 17]. Extracted ion chromatographs (EICs) from a standard solution (Figure 1(a)), a SZL sample (Figure 1(b)), and an SZL-medicated serum sample (Figure 1(c)) of the targeted analytes are shown in Figure 1. As seen from this figure, (i) according to the analytical methods used in this experiment, four target compounds had symmetrical chromatographic peak shapes. Albiflorin may not have been detected because of its low peak signal similar to noise. (ii) The chromatographic separation of various target compounds was achieved. (iii) There were no significant differences in retention time or chromatographic peak shape among SZL, SZL-medicated serum samples, and standard solution. Finally, the results showed that the SZL-medicated serum contained paeoniflorin, liquiritin, cinnamic acid, and glycyrrhizic acid (Table 2), indicating that the SZL entered the gastrointestinal tract of rats by gavage and entered the blood, thereby playing a therapeutic role.

### 3.2. STZ-Induced OLN-93 Cell Injury Was Established to Mimic the Pathological Changes of Myelin Sheath of AD

**Figure 2:**
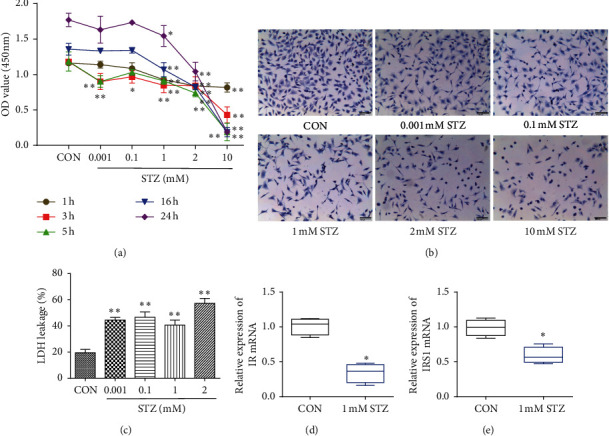
An in vitro mode of STZ-induced OLN-93 cell injury was established. (a) The cell viability of OLN-93 cells treated with STZ (0.001 mM–10 mM) for 1–24 h was detected by CCK-8. Each point represents the mean ± SD of *n* = 6 experiments. (b) Morphological changes of OLN-93 cells under microscope (scale = 100 *μ*m) after treatment with STZ at different concentrations for 3 hours was observed. (c) The relative LDH leakage of OLN-93 cells was determined after treatment with STZ at different concentrations for 3 h. Each point represents the mean ± SD of *n* = 6 experiments. Changes in IR (d) and IRS1 (e) mRNA transcription levels in OLN-93 cells after STZ treatment for 3 h were observed by qPCR. Mann–Whitney test was used to compare the difference of IR and IRS1 mRNA relative transcript level between control and STZ group. Each point represents the median (IQR) of *n* = 4 experiments. ^*∗*^*P* < 0.05 and ^*∗∗*^*P* < 0.01, significantly different from control group. IQR means interquartile range.

STZ-induced OLN-93 cell injury was established following previous experimental methods [18–21] and slightly improved according to the specific conditions of this experiment. First, the viability of OLN-93 cells incubated with different concentrations of STZ (0.001 mM–10 mM) diluted with serum-free DMEM was detected at different time points (1 h, 3 h, 5 h, 16 h, and 24 h). As shown in Figure 2(a), the cell viability of OLN-93 cells treated with 0.001 mM–10 mM STZ for 3 h was significantly lower than that in the control group (*P* < 0.01 or *P* < 0.05). When treated with 1 mM–10 mM STZ for 1 h, 5 h, 16 h, and 24 h or treated with 0.001 mM STZ for 5h, the cell viability was lower than that of the control group (*P* < 0.01 or *P* < 0.05). Second, we observed the effect of STZ on the morphology of OLN-93 cells. OLN-93 cells were seeded into a 12-well plate at a density of 1 × 10^5^/ml in 1 ml medium, and the morphology of the cells was observed under microscope after incubation with different concentrations of STZ for 3 hours. As shown in Figure 2(b), OLN-93 cells in the control group adhered well to the wall, and the cell body was long fusiform, with 2-3 protrusions in the cell body. Treated with STZ at increasing concentrations, the cell body gradually became thinner or even fragmented. The nucleus was pyknotic, which was significantly different from the control group. Third, the cytotoxicity of OLN-93 cells incubated with 0.001 mM–2 mM STZ for 3 h was detected. As shown in Figure 2(c), the relative LDH leakage can reflect cell cytotoxicity. When treated with 0.001 mM–2 mM STZ for 3 h, the LDH leakage increased gradually (*P* < 0.01) compared with that in the control group. Finally, OLN-93 cells were seeded at a density of 1 × 10^5^/ml into a 6-well cell culture plate in 2.5 ml medium, and qPCR was used to detect the transcript level of IR and IRS1 mRNA in OLN-93 cells. As shown in Figures 2(d) and 2(e), compared with the control group, the transcript level of IR (Figure 2(d)) and IRS1 (Figure 2(e)) mRNA was significantly decreased after 1 mM STZ treatment for 3 h (*P* < 0.05).

### 3.3. SZL-Medicated Serum-Increased Cell Viability of OLN-93 Cells

Since any drug, including monomers and active ingredients in traditional Chinese medicine in medicated serum, can be toxic to cells at a specific time and concentration. Firstly, we investigated whether SZL-medicated serum and donepezil were cytotoxic for normal OLN-93 cells. As shown in Supplementary Figure 1. After incubating for 16–48 h, 2.5%∼20% of SZL-medicated serum and 0.01–1 *μ*M of donepezil have no toxic effect on the normal cells.

**Figure 3:**
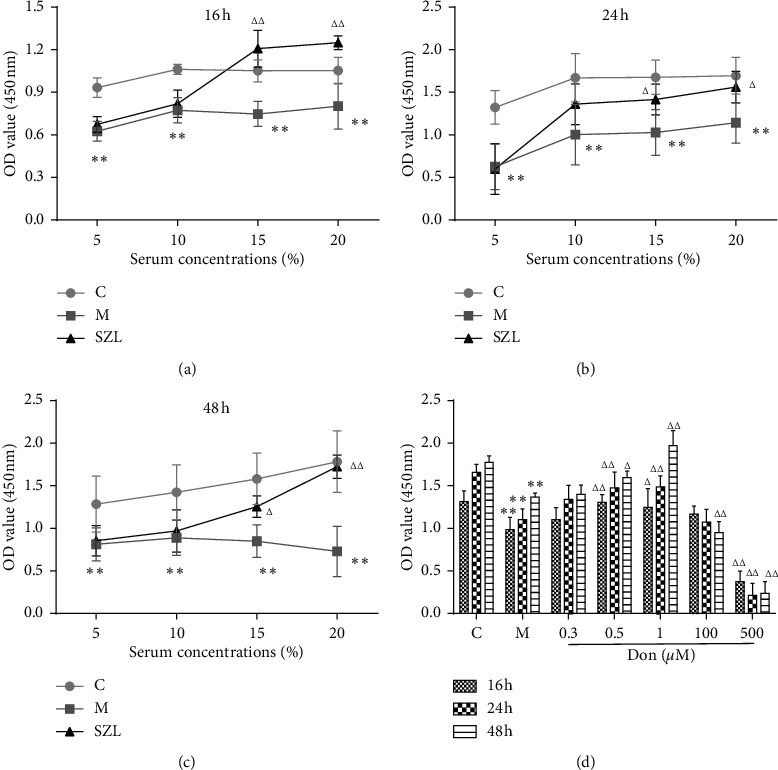
Effect of SZL-medicated serum (a–c) and donepezil (d) on cell viability of STZ-induced injury of OLN-93 cells. C, control group; M, model group; SZL, SZL-medicated serum group; Don, donepezil group. ^*∗*^*P* < 0.05 and ^*∗∗*^*P* < 0.01, significantly different from control group; ^Δ^*P* < 0.05 and ^ΔΔ^*P* < 0.01, significantly different from model group. Each point represents the mean ± SD of *n* = 6 experiments.

Furthermore, we explored the optimal concentration and time of SZL-medicated serum and donepezil to protect injured cells. After injuring OLN-93 cells with 1 mM STZ for 3 hours, the medium was absorbed and the cells were washed with PBS twice. The culture medium was changed according to the group conditions. The SZL-medicated serum was diluted to different concentrations (5%, 10%, 15%, and 20%) with DMEM and added to the corresponding group. Blank serum with a corresponding concentration was dropped into the control and model groups. Donepezil was diluted into five concentrations of 0.3 *μ*M, 0.5 *μ*M, 1 *μ*M, 100 *μ*M, and 500 *μ*M and added to the corresponding experimental group. Then, 100 *μ*l/well DMEM was added to the control group and the model group. As shown in Figures 3(a)–3(c), compared with the control group, the OD value of the model group decreased significantly at 16 h, 24 h, and 48 h (*P* < 0.01), indicating that the cells were damaged. Compared with the model group, 15% and 20% SZL-medicated serum significantly increased the OD value at 16 h, 24 h, and 48 h (*P* < 0.01 or *P* < 0.05), indicating that the cell damage was recovered. As shown in Figure 3(d), compared with the control group, the OD value of the model group was significantly decreased (*P* < 0.01). Compared with the model group, the OD value of the 0.5 *μ*M and 1 *μ*M donepezil groups increased significantly at 16 h, 24 h, and 48 h (*P* < 0.01 or *P* < 0.05). However, when the concentration of donepezil reached 500 *μ*M, the OD value was significantly lower (*P* < 0.01) than the model group at these three time points. At the same time, the OD value of the 100 *μ*M donepezil group was also significantly lower than that of the model group at 48 h (*P* < 0.01).

Combining the results of Figure 3 and Supplementary Figure 1, we selected 15% SZL-medicated serum and 0.5 *μ*M donepezil for the following experiment.

### 3.4. SZL-Medicated Serum-Increased Expression of PI3K, Akt, p-Akt, mTOR, and p-mTOR Proteins in STZ-Injured OLN-93 Cells

**Figure 4:**
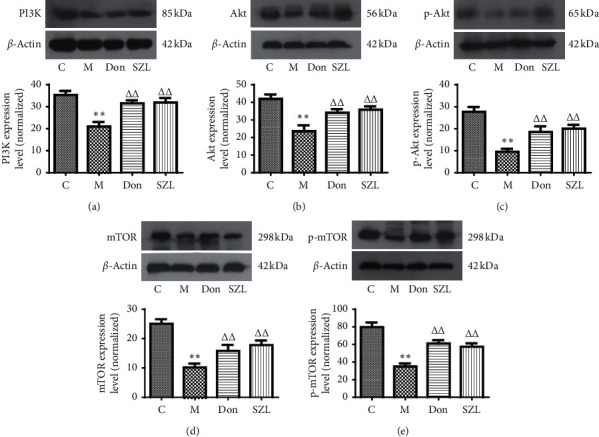
Effect of SZL-medicated serum on expression of PI3K (a), Akt (b), p-Akt (c), mTOR (d), and p-mTOR (e) proteins in injured OLN-93 cells. C, control group; M, model group; Don, donepezil group; SZL, SZL-medicated serum group. ^*∗∗*^*P* < 0.01, significantly different from control group; ^ΔΔ^*P* < 0.01, significantly different from model group. Each point represents the mean ± SD of *n* = 6 experiments.

Studies have shown that the PI3K/Akt-mTOR pathway plays an important role in regulating oligodendrocyte differentiation and myelination [5]. After OLN-93 cells were damaged by 1 mM STZ for 3 h, the culture medium was changed using the following groups: (1) the control group received serum-free DMEM + 15% blank serum, (2) the model group received serum-free DMEM + 15% blank serum, (3) the SZL group received serum-free DMEM + 15% SZL-medicated serum, and (4) the donepezil group received serum-free DMEM + 0.5 *μ*M donepezil + 15% blank serum. OLN-93 cells in each group were incubated in 37 °C in a 5% CO_2_ atmosphere for 24 h. Western blot and qPCR were used to detect the effect of SZL-medicated serum on the expression of the PI3K/Akt-mTOR pathway and myelin-related proteins and mRNA in STZ-injured OLN-93 cells. As shown in Figure 4, the expression of PI3K, Akt, p-Akt, mTOR, and p-mTOR proteins in the model group was significantly lower than in the control group (*P* < 0.01). Incubating in 0.5 *μ*M donepezil or 15% SZL-medicated serum can significantly increase the expression of PI3K, Akt, p-Akt, mTOR, and p-mTOR proteins, which were significantly different from the model group (*P* < 0.01).

### 3.5. SZL-Medicated Serum-Increased Expression of MBP, MOG, and PLP Proteins in STZ-Injured OLN-93 Cells

**Figure 5:**
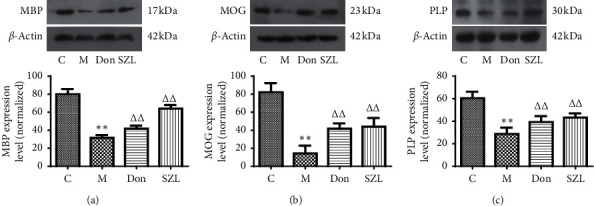
Effect of SZL-medicated serum on expression of MBP (a), MOG (b), and PLP (c) proteins in injured OLN-93 cells. C, control group; M, model group; Don, donepezil group; SZL, SZL-medicated serum group. ^*∗∗*^*P* < 0.01, significantly different from control group; ^ΔΔ^*P* < 0.01, significantly different from model group. Each point represents the mean ± SD of *n* = 6 experiments.

PLP and MBP constitute the majority of myelin total protein [22], and MOG was located on the surface of myelin, making it an excellent antibody target [23]. As shown in Figure 5, the expression of MBP, MOG, and PLP proteins in the model group was significantly lower than in the control group (*P* < 0.01). The 15% SZL-medicated serum and the 0.5 *μ*M donepezil significantly increased the expression of these proteins (*P* < 0.01).

### 3.6. SZL-Medicated Serum-Increased Expression of Akt, mTOR, and MBP mRNA in STZ-Injured OLN-93 Cells

**Figure 6:**
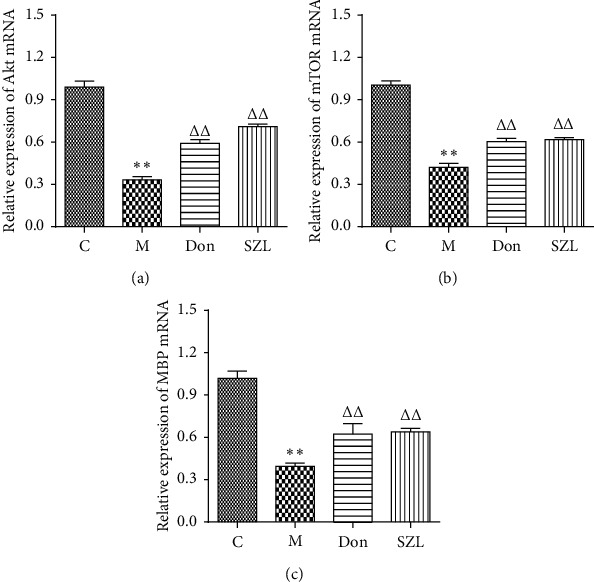
Effect of SZL-medicated serum on expression of Akt (a), mTOR (b), and MBP (c) mRNA in injured OLN-93 cells. C, control group; M, model group; Don, donepezil group; SZL, SZL-medicated serum group. ^*∗∗*^*P* < 0.01, significantly different from control group; ^ΔΔ^*P* < 0.01, significantly different from model group. Each point represents the mean ± SD of *n* = 6 experiments.

As shown in Figure 6, the expression of Akt, mTOR, and MBP mRNA in the model group was significantly lower than in the control group (*P* < 0.01). The 0.5 *μ*M donepezil and 15% SZL-medicated serum significantly increased the expression of Akt, mTOR, and MBP mRNA, which were significantly different from the model group (*P* < 0.01).

### 3.7. PI3K Pathway Inhibitor LY294002 Inhibited Protective Effect of SZL-Medicated Serum on STZ-Injured OLN-93 Cells

We confirmed in the above experiments that SZL-medicated serum can protect myelin by improving the activity of the PI3K/Akt-mTOR signalling pathway after injury. To further confirm the involvement of the PI3K/Akt-mTOR signalling pathway, we blocked the PI3K/AKT signalling pathway using the PI3K pathway inhibitor LY294002 and then inhibited the mTOR downstream phosphorylation target with the mTOR inhibitor rapamycin. After OLN-93 cells were damaged by 1 mM STZ for 3 h, the culture medium was changed using the following groups: (1) a control group with serum-free DMEM + 15% blank serum, (2) a model group with serum-free DMEM + 15% blank serum, (3) a model + LY294002/rapamycin group with serum-free DMEM + 15% blank serum + 20 *μ*M LY294002/rapamycin, (4) a donepezil group with serum-free DMEM + 0.5 *μ*M donepezil + 15% blank serum, (5) a donepezil + LY294002/rapamycin group with serum-free DMEM + 0.5 *μ*M donepezil + 15% blank serum + 20 *μ*M LY294002/rapamycin, (6) a SZL group with serum-free DMEM + 15% SZL-medicated serum, and (7) a SZL + LY294002/rapamycin group with serum-free DMEM+15% SZL-medicated serum + 20 *μ*M LY294002/rapamycin. OLN-93 cells in each group were incubated in a 37°C, 5% CO_2_ atmosphere for 24 h.

**Figure 7:**
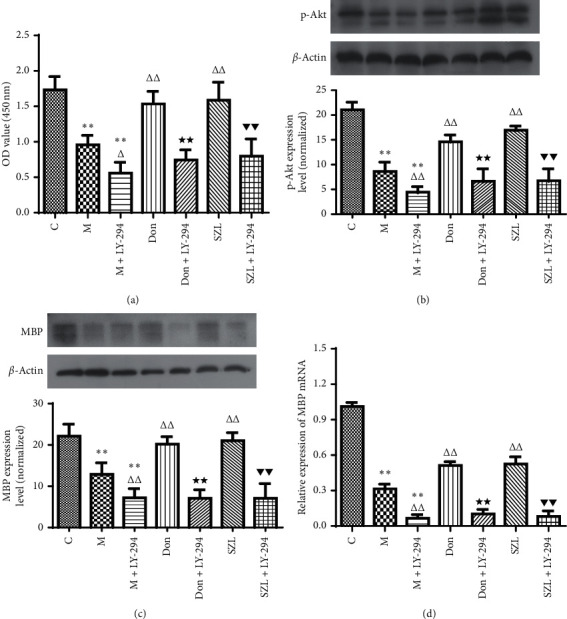
The role of LY294002 on protective effect of SZL-medicated serum on STZ-injured OLN-93 cells. (a) Effect of LY294002 on SZL-medicated serum-increased cell viability of injured OLN-93 cells. Effect of LY294002 on SZL-medicated serum-induced up-regulation of p-Akt (b) and MBP (c, d) in injured OLN-93 cells; C, control group; M, model group; M + LY-294, model + LY294002 group; Don, donepezil group; Don + LY-294, donepezil + LY294002 group; SZL, SZL-medicated serum group; SZL + LY-294, SZL-medicated serum + LY294002 group. ^*∗∗*^*P* < 0.01, significantly different from control group. ^Δ^*P* < 0.05 and ^ΔΔ^*P* < 0.01, significantly different from model group. ^★★^*P* < 0.01, significantly different from donepezil group; ^▼▼^*P* < 0.01, significantly different from SZL-medicated serum group. Each point represents the mean ± SD of *n* = 6 experiments.

As shown in Figure 7(a), compared with the control group, the OD value in the model group was significantly decreased (*P* < 0.01). Compared with the model group, the OD values in the SZL group and the donepezil group were significantly increased (*P* < 0.01). Compared with the model group, the OD value in the model + LY294002 group was significantly decreased (*P* < 0.05). The donepezil + LY294002 group had significantly reduced OD value compared to the donepezil group (*P* < 0.01). Similarly, compared with the SZL group, the SZL + LY294002 group had significantly reduced OD value (*P* < 0.01). As shown in Figures 7(b)–7(d), compared with the control group, the expression of p-Akt protein, MBP protein, and MBP mRNA in the model group was significantly decreased (*P* < 0.01). Compared with the model group, the expression of p-Akt protein, MBP protein, and MBP mRNA in the SZL group and the donepezil group was significantly increased (*P* < 0.01). Compared with the model group, the expression of p-Akt protein, MBP protein, and MBP mRNA in the model + LY294002 group was significantly decreased (*P* < 0.01). The donepezil + LY294002 group had significantly reduced p-Akt protein, MBP protein, and MBP mRNA expression compared to the donepezil group (*P* < 0.01). Similarly, compared with the SZL group, the SZL + LY294002 group had significantly reduced expression of p-Akt protein, MBP protein, and MBP mRNA (*P* < 0.01).

### 3.8. Rapamycin Inhibited Protective Effect of SZL-Medicated Serum on STZ-Injured OLN-93 Cells

**Figure 8:**
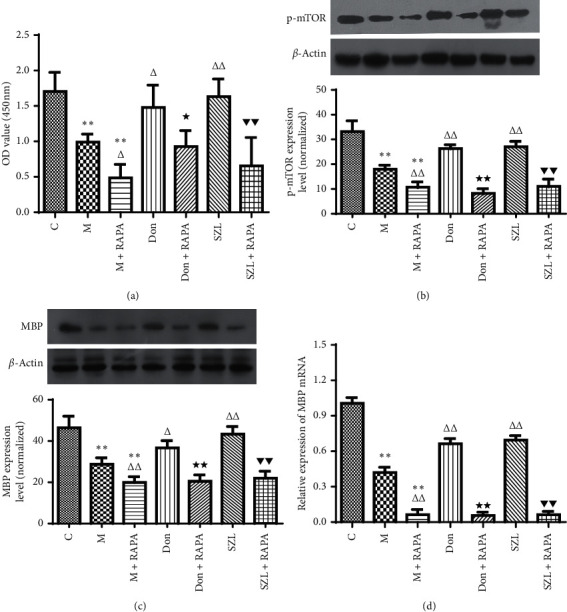
The role of rapamycin on protective effect of SZL-medicated serum on STZ-injured OLN-93 cells. (a) Effect of rapamycin on SZL-medicated serum-increased cell viability of injured OLN-93 cells. Effect of rapamycin on SZL-medicated serum-induced upregulation of p-mTOR (b) and MBP (c, d) in injured OLN-93 cells. C, control group; M, model group; M + RAPA, model + rapamycin group; Don, donepezil group; Don + RAPA, donepezil + rapamycin group; SZL, SZL-medicated serum group; SZL + RAPA, SZL-medicated serum + rapamycin group. ^*∗∗*^*P* < 0.01, significantly different from control group; ^Δ^*P* < 0.05 and ^ΔΔ^*P* < 0.01, significantly different from model group; ^★^*P* < 0.05 and ^★★^*P* < 0.01, significantly different from donepezil group; ^▼▼^*P* < 0.01, significantly different from SZL-medicated serum group. Each point represents the mean ± SD of *n* = 6 experiments.

As shown in Figure 8(a), compared with the control group, the OD value in the model group was significantly decreased (*P* < 0.01). Compared with the model group, the OD values in the SZL group and the donepezil group were significantly increased (*P* < 0.01 or *P* < 0.05). Compared with the model group, the OD value in the model + rapamycin group was significantly decreased (*P* < 0.05). The donepezil + rapamycin group had significantly reduced OD value compared to the donepezil group (*P* < 0.05). Similarly, compared with the SZL group, the SZL + rapamycin group had significantly reduced OD value (*P* < 0.01). As shown in Figures 8(b)–8(d), compared with the control group, the expression of p-mTOR protein, MBP protein, and MBP mRNA in the model group was significantly decreased (*P* < 0.01). Compared with the model group, the expressions of p-mTOR protein, MBP protein, and MBP mRNA in the SZL group and the donepezil group were significantly increased (*P* < 0.01 or *P* < 0.05). Compared with the model group, the expressions of p-mTOR protein, MBP protein, and MBP mRNA in the model + rapamycin group was significantly decreased (*P* < 0.01). The donepezil + rapamycin group had significantly reduced p-mTOR protein, MBP protein, and MBP mRNA expressions compared to the donepezil group (*P* < 0.01). Similarly, compared with the SZL group, the SZL + rapamycin group had significantly reduced expressions of p-mTOR protein, MBP protein, and MBP mRNA (*P* < 0.01).

## 4. Discussion

The myelin sheath consists of multilayer lipids and proteins, including myelin basic protein (MBP), proteolipid protein (PLP), and myelin oligodendrocyte glycoprotein (MOG). These membrane-related proteins are the key to maintaining normal physiological function of the myelin sheath. Studies have shown that mature OLs transplantation into the rat brain cannot effectively repair the damaged myelin sheath [24]. OPCs, on the other hand, can aggregate and migrate, reactivating into the cell cycle when the myelin sheath is damaged. Richter-Landsberg successfully established an immortalized cell line, OLN-93, in 1996 [25]. This cell line was spontaneously transformed from cultured rat glial cells in vitro and could express proteins such as MOG, MBP, and PLP, which appeared in the mature stage of myelin sheath development, without losing OPCs' proliferation ability [26]. This cell line was stable in the stage between pre-OLGs and immature OLGs. Thus, we selected this cell line.

STZ, a glucosamine derivative of nitrosourea, was often used to study diabetes and the relationship between diabetes and AD. *In vivo*, lateral ventricular injection of STZ can induce AD-like behavioural, cognitive, and neuropathological changes in rats or mice. This method was considered to be a reliable, standard, and repeatable method for establishing experimental models of AD [27]. In addition, STZ can reduce brain glucose utilization [28], decrease the expression of insulin receptors (IRs) in the cortex and hippocampus, disrupt insulin synthesis, and cause disorders in insulin-signalling pathways [29]. PI3K/AKT is an important and classical pathway among insulin-signalling pathways. IRS plays an important role in the insulin-signalling pathway. After IRS1 activation, it can support the survival of mature neurons through the PI3K/Akt pathway [30]. Previous studies by our research group showed that STZ disrupted the insulin-signalling pathway, leading to mitochondrial dysfunction and inhibiting cell survival and growth in SH-SY5Y cells [31]. Besides, SZL had a definite protective effect on the myelin sheath and synapses of AD mice that received STZ injected into the lateral ventricle (results to be published). Incubating OLN-93 cell with STZ, we simulated an insulin signal-impairment. CCK-8 analysis showed that STZ significantly reduced cell viability of OLN-93 cells and increased LDH leakage. HE staining revealed that STZ could damage cell morphology. QPCR demonstrated that STZ decreased expression of IR and IRS1 mRNA. The results raise the possibility that STZ may cause the disturbance of PI3K/Akt pathway and lead to cell damage by damaging the expression of IR and IRS1.

The processes of myelin formation and myelin regeneration are strictly regulated by multiple coordinated signal transduction pathways, such as the Wnt/*β*-catenin, PI3K/Akt-mTOR, and ERK/MAPK pathways. Among them, the PI3K/Akt-mTOR pathway plays an important role in regulating the differentiation of oligodendrocytes and the formation of the myelin sheath [5]. Activation of Akt in oligodendrocytes reduced axonal sheath defects caused by cholesterol deficiency [32]. The mTOR inhibitor, rapamycin, can significantly reduce the levels of myelin gene transcription and myelin proteins in the mouse brain [33]. The PI3K/Akt pathway in the brain of AD patients was downregulated, and upregulation of the PI3K/Akt pathway can significantly reduce Tau-induced neurotoxicity and A*β* deposition by activating the PI3K/Akt pathway [34, 35]. In our experiment, STZ disturbed the PI3K/Akt-mTOR signalling pathway in OLN-93 cells, and the downregulation of myelin sheath-related proteins was also found. After STZ injury, SZL-medicated serum can improve the activity of the PI3K/Akt-mTOR signalling pathway and play a protective role for the myelin. Our results also showed that PI3K inhibitor, LY294002, inhibited the promotion of cell viability, p-Akt, and MBP expression by SZL-medicated serum, and rapamycin inhibited the promotion of cell viability, p-mTOR, and MBP expression by SZL-medicated serum. Thus, the protective effect of SZL-medicated serum on oligodendrocytes and myelin sheath may be through the PI3K/Akt-mTOR signal transduction pathway.

Donepezil is one of the four drugs approved by US FDA for the treatment of AD. It is a highly selective acetylcholinesterase inhibitor, mainly aimed at the cholinergic system. However, some studies have found that donepezil can play a neuroprotective mechanism by cholinergic system-independent way [36–42]. Donepezil was selected as the positive control chemical in this study based on the following two points. Firstly, donepezil has the effect of promoting the differentiation of OPCs into mature OLs. Other AChEIs, such as Huperzine A, Kapalatin, and Tacrine, have no such effect. More significantly, donepezil not only promotes myelin formation in OPC-DRG neuron cocultures in vitro, but also promotes myelin repair in vivo [36]. Donepezil can increase the mRNA expression of myelin-related genes (such as PLP, MAG, MBP, CNPase, and MOG) [37]. Secondly, donepezil can play a therapeutic role through PI3K/Akt and related signalling pathways. Donepezil protects neurons from moderate glutamate neurotoxicity through the nAChR-PI3K-Akt and MAPK signalling pathways [38, 39]. The neuroprotective effect of donepezil on A*β*_25-35_-injured SH-SY5Y is closely related to the PI3K-Akt pathway [40]. Donepezil inhibits bradykinin-induced increase of reactive oxygen levels in astrocytes and related inflammatory responses through nAChR and PI3K-Akt pathway [41], and the anti-inflammatory activity of donepezil was also associated with PI3K/Akt/NF-*κ*B [42]. Therefore, we chose donepezil as a positive control chemical. At the same time, in our experiment, we also verified the effect of donepezil. Donepezil can not only improve cell activity and increase myelin sheath-related protein and mRNA expression, but also enhance the activity of PI3K/Akt-mTOR signalling pathway. But donepezil as a positive control chemical is still controversial and needs further study.

In our experiments, both SZL-medicated serum and donepezil can protect cells from STZ-caused damage, increase OLN-93 cell viability in a dose- and time-dependent manner, and enhance the activity of PI3K/Akt-mTOR signalling pathway. However, the safety of drugs is a very important issue. SZL-medicated serum has no toxic effect on OLN-93 cell. However, donepezil was toxic to cells at concentrations above 100 *μ*M. The relevant mechanism needs further investigation.

Our UHPLC-MRM-MS/MS results showed that SZL-medicated serum contained paeoniflorin, liquiritin, cinnamic acid, and glycyrrhizic acid. The active constituents of SZL, paeoniflorin, and glycyrrhizic acid were confirmed to have a definite therapeutic effect on AD in previous study. Paeoniflorin can play a therapeutic role in STZ-induced cognitive deficits in mice by improving insulin signal transduction [43]. Paeoniflorin also alleviates okadinic acid induced damage of SH-SY5Y cells by interfering with the Akt/GSK-3*β*-related pathway and alleviates the microtubule structure system stress response induced by okadinic acid [44]. Glycyrrhizic acid had a significant effect on insulin resistance in rats [45]. It also exerts an anti-neurotoxic effect in the SH-SY5Y cell injury model induced by autophagy 6-hydroxydopamine and corticosterone [46]. Therefore, we can speculate that the protective effect of SZL on oligodendrocytes and myelin sheath was probably related to the absorption of these four active compounds in serum.

## 5. Conclusions

In summary, our data demonstrated that OLN-93 cells damaged by STZ can mimic the characteristics and symptoms of AD. The PI3K/Akt-mTOR signalling pathway was damaged by STZ, which led to inhibition of cell survival and growth. SZL plays a protective role on OLN-93 cell by stimulating PI3K/Akt-mTOR signalling pathway-related protein and mRNA expression. In addition, the important role of PI3K/Akt-mTOR signalling pathway was confirmed by using inhibitors LY294002 and rapamycin. Since TCM has multicomponent and multitarget effects, there may be more mechanisms to be further explored. Our present study will facilitate the research of AD.

## Figures and Tables

**Table 1:** qPCR primer sequences for IR, IRS1, Akt, mTOR, and MBP.

Gene	Forward (5′ ⟶ 3′)	Reverse (5′ ⟶ 3′)
IR	AGAGGTGGGCAATGTGACAG	ATGCGGTACCCAGTGAAGTG
IRS1	ACCATCAGCAAGCAGGTCATTGT	CGGGTCCTCCACTTCACGAC
Akt	GGCAGGAGGAGGAGACGATGG	TTCATGGTCACACGGTGCTTGG
mTOR	AGAACCTGGCTCAAGTACGC	AGGATGGTCAAGTTGCCGAG
MBP	CTATAAATCGGCTCACAAGG	TGTGGGCGACTTCATCCT

**Table 2:** Metabolite concentrations in SZL and SZL-medicated serum.

Name	*n*	SZL (*μ*mol/L)	SZL-medicated serum (nmol/L)
Albiflorin	4	2619.21 ± 129.13	ND
Cinnamic acid	4	1168.93 ± 55.36	1005.60 ± 51.06
Glycyrrhizic acid	4	7564.53 ± 1079.91	32945.61 ± 31235.12
Liquiritin	4	1027.11 ± 50.39	38.96 ± 10.90
Paeoniflorin	4	6938.82 ± 453.27	72.84 ± 20.14

ND indicates that the target compound in this sample was not detected.

## Data Availability

The data used to support the findings of this study are available from the corresponding author upon request.

## Abbreviations

AD: Alzheimer's disease
SZL: Shenzhiling oral liquid
STZ: Streptozotocin
IR: Insulin receptor
IRS: Insulin receptor substrate
MBP: Myelin basic protein
MOG: Myelin oligodendrocyte glycoprotein
PLP: Proteolipid protein
OLs: Oligodendrocyte-s
OPCs: Oligodendrocyte precursor cells
LDH: Lactate dehydrogenase
PI3K: Phosphatidylinositol 3-kinase
mTOR: Mammalian target of rapamycin.

## Supplementary Materials

Supplementary Materials Supplementary Figure 1: effect of SZL-medicated serum (A–C) and donepezil (D) on cell viability of normal OLN-93 cells.
