# Supplementary material for: Shenzhiling Oral Liquid Protects STZ-Injured Oligodendrocyte through PI3K/Akt-mTOR Pathway
Source: Evid Based Complement Alternat Med. 2020 Jul 24;2020:4527283. doi: 10.1155/2020/4527283 (PMC7396001; doi:10.1155/2020/4527283)
Supplement: Supplementary Materials — Supplementary Figure 1: effect of SZL-medicated serum (A–C) and donepezil (D) on cell viability of normal OLN-93 cells. [file 4527283.f1.docx]

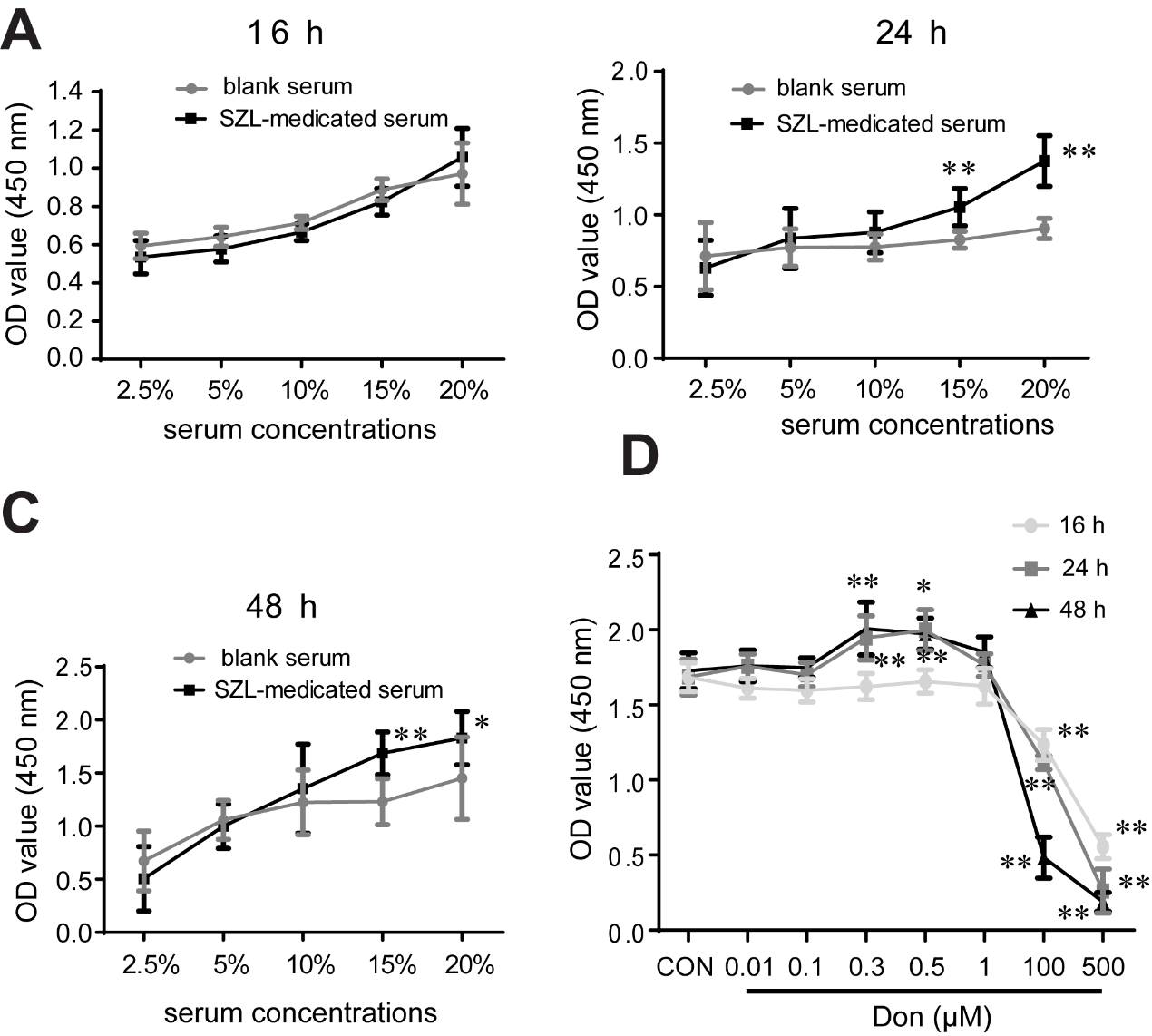


Supplementary Figure 1: Effect of SZL-medicated serum (A-C) and donepezil (D) on cell viability of normal OLN-93 cells.

* *P*<0.05, ** *P*<0.01, significantly different from blank serum group (A-C) or control group (D). Each point represents the mean ± SD of n=6 experiments.

After 24 h of OLN-93 cell culture, the medium was changed according to the following group. SZL-medicated serum was diluted with DMEM to different concentrations (2.5%, 5%, 10%, 15%, 20%) and dropped into the corresponding experimental groups. Blank serum with corresponding concentration was dropped into the control group. Donepezil was diluted to seven concentrations of 0.01 μM, 0.1 μM, 0.3 μM, 0.5 μM, 1 μM, 100 μM, and 500 μM and added to the corresponding experimental group, and 100 μl/ well DMEM was added to the control group. Each group was cultured in 37℃ and a 5% CO_2_ atmosphere to detect cell activity for 16 h, 24 h and 48 h. A-C: Compared with the blank serum group, the OD value of OLN-93 cells incubated with 2.5%~20% SZL-medicated serum for 16 h were not significantly different (*P*>0.05). After the cells were incubated with 15% and 20% SZL-medicated serum for 24 h and 48 h, the OD value was significantly increased (*P*<0.01 or *P*<0.05), suggesting that the viability of OLN-93 cells increased. D: The OD value of OLN-93 cells incubated with 0.3 μM or 0.5 μM of Donepezil for 24 h and 48 h significantly increased (*P*<0.01 or *P*<0.05). Nevertheless, compared with the control group, the OD value of OLN-93 cells incubated with 100 μM or 500 μM of Donepezil for 16 h, 24 h and 48 h significantly reduced (*P*<0.01).
